# Decentral gene expression analysis: analytical validation of the Endopredict genomic multianalyte breast cancer prognosis test

**DOI:** 10.1186/1471-2407-12-456

**Published:** 2012-10-05

**Authors:** Ralf Kronenwett, Kerstin Bohmann, Judith Prinzler, Bruno V Sinn, Franziska Haufe, Claudia Roth, Manuela Averdick, Tanja Ropers, Claudia Windbergs, Jan C Brase, Karsten E Weber, Karin Fisch, Berit M Müller, Marcus Schmidt, Martin Filipits, Peter Dubsky, Christoph Petry, Manfred Dietel, Carsten Denkert

**Affiliations:** 1 Sividon Diagnostics GmbH, Nattermannallee 1, 50829, Cologne, Germany; 2 Institute of Pathology, Charité Hospital, Campus Mitte, Berlin, Germany; 3 Department of Gynecology and Obstetrics, University of Mainz, Mainz, Germany; 4 Department of Medicine I, Medical University of Vienna, Vienna, Austria; 5 Department of Surgery, Medical University of Vienna, Vienna, Austria

**Keywords:** Breast cancer, Prognostic multigene expression test, Analytical validation, PCR, Pathology

## Abstract

**Background:**

EndoPredict (EP) is a clinically validated multianalyte gene expression test to predict distant metastasis in ER-positive, HER2-negative breast cancer treated with endocrine therapy alone. The test is based on the combined analysis of 12 genes in formalin-fixed, paraffin-embedded (FFPE) tissue by reverse transcription-quantitative real-time PCR (RT-qPCR). Recently, it was shown that EP is feasible for reliable decentralized assessment of gene expression. The aim of this study was the analytical validation of the performance characteristics of the assay and its verification in a molecular-pathological routine laboratory.

**Methods:**

Gene expression values to calculate the EP score were assayed by one-step RT-qPCR using RNA from FFPE tumor tissue. Limit of blank, limit of detection, linear range, and PCR efficiency were assessed for each of the 12 PCR assays using serial samples dilutions. Different breast cancer samples were used to evaluate RNA input range, precision and inter-laboratory variability.

**Results:**

PCR assays were linear up to C_q_ values between 35.1 and 37.2. Amplification efficiencies ranged from 75% to 101%. The RNA input range without considerable change of the EP score was between 0.16 and 18.5 ng/μl. Analysis of precision (variation of day, day time, instrument, operator, reagent lots) resulted in a total noise (standard deviation) of 0.16 EP score units on a scale from 0 to 15. The major part of the total noise (SD 0.14) was caused by the replicate-to-replicate noise of the PCR assays (repeatability) and was not associated with different operating conditions (reproducibility). Performance characteristics established in the manufacturer’s laboratory were verified in a routine molecular pathology laboratory. Comparison of 10 tumor samples analyzed in two different laboratories showed a Pearson coefficient of 0.995 and a mean deviation of 0.15 score units.

**Conclusions:**

The EP test showed reproducible performance characteristics with good precision and negligible laboratory-to-laboratory variation. This study provides further evidence that the EP test is suitable for decentralized testing in specialized molecular pathological laboratories instead of a reference laboratory. This is a unique feature and a technical advance in comparison with existing RNA-based prognostic multigene expression tests.

## Background

EndoPredict (EP) is a multigene assay which predicts the risk of distant metastasis in ER+/HER2- breast cancer and identifies a subgroup of patients who have an excellent prognosis if treated with endocrine therapy alone [1]. The test is based on the assessment of expression of 8 informative genes, 3 reference genes, and one gene to measure the presence of genomic DNA in RNA from formalin-fixed, paraffin-embedded (FFPE) tissue from biopsies or surgical specimens using reverse transcription-quantitative real-time PCR (RT-qPCR) [1-3]. Relative gene expression levels are used to calculate the EndoPredict score (EP score) ranging from 0 to 15. Patients with a score below or equal to 5 are classified as low risk for distant recurrence under endocrine therapy, those with a score above 5 as high risk.

**Figure 1:**
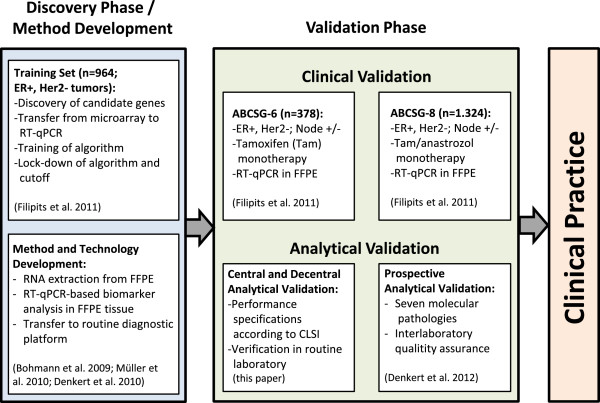
**Translation of the EndoPredict multigene expression test from research laboratory to clinical practice.** Workflow of sequential discovery and clinical as well as analytical validation is shown.

Translation of the EP test from research laboratory to clinical practice covered the necessary steps for development of a laboratory test (Figure 1). This included method development for standardized RNA extraction from FFPE tissue [4-6] and transfer of RT-qPCR assays to a certified routine diagnostic platform [2,7] as well as a discovery phase with biomarker identification and training of an algorithm in a multicenter cohort [1]. Following discovery the pre-defined, locked-down EP score was clinically validated in two separate cohorts from the two randomized clinical trials ABCSG-6 (n=378) and ABCSG-8 (n=1324) [1]. Moreover, it has been shown that the EP score provided prognostic information on the risk of distant metastasis of breast cancer patients beyond clinic-pathological parameters such as ki-67 and quantitative ER immunohistochemistry [1].

Besides EndoPredict, other prognostic multigene expression tests for patients with breast cancer like MammaPrint [8], Oncotype DX [9,10], or PAM50 [11,12] are commercially available. However, all these different tests can only be performed in reference laboratories. In contrast, EndoPredict is suitable for decentralized testing in specialized molecular pathological laboratories as recently shown in a prospective analytical proficiency testing program with seven different molecular pathological laboratories [2].

The aim of this study was a comprehensive analytical validation of the EP test to complete development before implementation in clinical practice. Analytical validation of multianalyte assays is still a challenge, as these types of assays require a more complex evaluation of the performance characteristics compared with single analyte assays in order to assure reliable performance in the clinical routine. Adequate performance evaluation includes the control of the process from the acquisition of the tumor samples and isolation of the RNA to the assessment of each single analyte as well as the combination of the single results to a comprehensive score by an algorithm. Moreover, guidelines for analytical validation of multianalyte genomic assays are rare. Here, we analytically validated the EndoPredict multianalyte gene expression assay according to the adapted guideline MM17-A of the Clinical and Laboratory Standards Institute (CLSI) addressing the analytical validation of nucleic acid-based qualitative and semiquantitative multiplex assays [13]. Moreover, the performance characteristics of the assay were verified in an independent molecular pathological laboratory to confirm that the test meets its specifications when used in a routine diagnostic laboratory.

## Methods

### Reference and Testing Materials

Nucleic acids for test development and validation were selected based on the specific purpose of the respective analytical performance characteristics to be tested. Sample material was comparable to the specimen used in the clinical testing, i.e. DNA-free total RNA or genomic DNA from FFPE tissue which is fragmented to nucleic acid pieces by formalin-fixation [5,14]. Details about reference nucleic acids are described in supplemental data (see Additional file 1). In brief, for assessment of limit of detection (LoD), linear range, and efficiency of the single PCR assays large pools of control RNA and control DNA from different FFPE tumor blocks were generated and used for the experiments [15]. For the precision studies, three tumor specimens classified by the EP as low risk, high risk or close to the decision point were selected. For the correlation study, ten tumor samples were chosen with EP scores spanning the larger part of the full score range. These ten tumor samples were different to the ten samples used in the recently published EndoPredict proficiency testing [2]. This study was carried out in compliance with the Helsinki Declaration and was approved by the Ethics Committee of the Charité Hospital (Ref. No. EA1/139/05, Amendment 2008).

As positive controls of RT-qPCR assays a standardized reference RNA (Stratagene qPCR Human Reference Total RNA, Agilent Technologies, Böblingen, Germany) and Human Genomic DNA (Roche Applied Bioscience, Mannheim, Germany) were tested on each plate.

### Isolation of RNA and DNA

Total RNA and DNA was extracted from FFPE tissue sections (10 μm) using a fully-automated silica-coated magnetic bead-based method in combination with a liquid handling robot (VERSANT Tissue Preparation System, Siemens Healthcare Diagnostics, Eschborn, Germany) as published previously [4-6].

The mean of C_q_ (quantification cycle) values of the EP reference genes *RPL37A*, *CALM2* and *OAZ1* was used as surrogate marker for mRNA yield following isolation. Concentration of total RNA was assessed using the QUANT-iT RIBOGREEN assay (Life Technologies, Darmstadt, Germany). For assessment of contamination with residual DNA in RNA preparations, an *HBB* gene-specific quantitative PCR was performed. Samples were considered to be substantially free of DNA when C_q_ values above 38 were detected. In case of DNA contamination samples were manually re-digested by DNase I treatment.

### Gene expression analysis using RT-qPCR

Expression of 8 genes-of-interest (*AZGP1, BIRC5, DHCR7, IL6ST, MGP, RBBP8, STC2, UBE2C*) and three reference genes (*CALM2, OAZ1, RPL37A*) as well as the amount of residual genomic DNA (*HBB*) were assessed by the EndoPredict assay (Sividon Diagnostics, Cologne, Germany) as previously described [1,2]. This assay is configured on a 96-well plate containing primers and FAM/TAMRA-labeled hydrolysis probes dried into the wells. Functional details about genes, data base accession numbers and sequences of primers and probes were published previously [1]. Gene expression was assessed by one-step RT-qPCR using the SuperScript III PLATINUM One-Step Quantitative RT-PCR System with ROX (Life Technologies, Darmstadt, Germany) according to manufacturer’s instructions in a VERSANT kPCR Molecular System (Siemens Healthcare Diagnostics, Eschborn, Germany) with 30 min at 50°C, 2 min at 95°C followed by 40 cycles of 15 sec at 95°C and 30 sec at 60°C. 20 μl reaction mix containing buffer, nucleotides, 4.5 mM Mg^2+^, enzymes and 1 μl sample RNA, respectively, were added to each well. The gene-specific reverse PCR primers were used as primers for reverse transcription. Since the *HBB*-specific assay did not target mRNA sequences the RT-qPCR protocol as described above could be used all the same.

For calculation of the EP score genes were measured in triplicates. This is mandatory to control for PCR imprecision and to enable outlier removal [1,2]. C_q_ values were calculated by the VERSANT kPCR Molecular System software using amplification-based thresholds following baseline correction according to manufacturer’s instructions. Detection of outliers, relative expression levels of each gene-of-interest ([GOI]; Δ*C*_*q*_(*GOI*) = 20 – *C*_*q*_ (*GOI*) + *C*_*q*_ (*CALM*2) + *C*_*q*_ (*OAZ1*) + *C*_*q*_ (*RPL37 A*)]/3) as well as EP scores were calculated as described previously using a web-based implementation to process analytical PCR results into EP scores which can be found at: http://forschung.medizin.uni-mainz.de/epreport/[1].

### Assessment of limit of blank (LoB), limit of detection (LoD), linear dynamic range and PCR efficiency

LoB, defined as the 5%-percentile of the distribution of C_q_-values measured in a blank sample without analyte, was calculated as described in supplemental data (see Additional file 1) [16]. LoD was defined as the amount of the reference RNA or DNA at which the C_q_ value is below the LoB with a probability of 95%. Since an absolute quantification of the 12 different targets in total RNA or DNA from FFPE tumors was not possible LoD was referred to the fold-dilution of the reference nucleic acid and to the respective C_q_ value as a surrogate for the amount of the individual analytes. For assessment of LoD and linear dynamic range, four independent series of 20 gravimetrically controlled serial 1:2 dilutions (log_2_) were generated from a pooled RNA sample (DNA sample for *HBB* PCR) from FFPE tissue resulting in 21 different concentrations [16-18]. Details about the dilution series and assessment of LoD and linear range are described in supplemental data (see Additional file 1). For each single PCR assay the linear dynamic range was determined by fitting a linear, quadratic, or cubic model. A maximum deviation from linearity of 1 Ct value was accepted. After assessing the linear dynamic range, the PCR efficiency was calculated by E = (2^-1/m^-1) x 100% where m is the slope of the linear regression model.

### Assessment of precision

The precision experiment was designed according to CLSI guidelines [13] and evaluated following ISO 5725-2 and NCCLS EP5-A2 [19,20]. The following variables were included: day (n=11), day time (n=2), PCR instrument (n=4), position of sample on 96-well EndoPredict plate (n=2), lot of EndoPredict plate (n=4), lot of enzyme/master mix (n=2), and operator (n=3). The experiment was performed during 28 calendar days including a 5 working days familiarization period at the beginning. Three different RNAs were used as test samples: One sample from a tumor with a low EP score (2.4), one with a high EP score (13.5) and one at the decision point between low and high risk (4.9). RNA was isolated from several sections and pooled for each tumor to have sufficient RNA for the whole precision experiment. In addition to the test samples, one quality control sample (Stratagene qPCR Human Reference Total RNA) was analyzed in each run.

For verification in the laboratory of Charité as a representative routine laboratory an abbreviated precision experiment with fewer variables was performed: day (n=5), day time (n=2), position of sample on 96-well EndoPredict plate (n=2), lot of enzyme/master mix (n=2), and operator (n=2).

Variable noise, replicate noise and total noise was calculated using univariate N-way analysis of variance (ANOVA) and indicated as standard deviations [18] as described in detail in supplemental data (see Additional file 1).

### Statistics

For EP scores 95% confidence intervals (CI) were calculated as described [1]. For comparison of EP test results between two different laboratories Pearson correlation coefficient (R^2^) was calculated and agreement of measurements were analyzed as described by Bland & Altman [21].

## Results

### Limit of blank, limit of detection, linear dynamic range and PCR efficiency

For each of the 12 genes the analytical performance of the RT-qPCR assays was assessed. For a type I error of 5% the LoB was at a C_q_ value of 40 for all genes (Table 1). The LoD of the 12 assays ranged from C_q_ 35.1 to 37.2 (Table 1; supplemental data Figure 1 [see Additional file 1]).

**Table 1:** LoB, LoD, linear dynamic range, PCR efficiency of the 12 PCR assays included in EndoPredict

**Gene**	**LoB**	**LoD [C**_**q**_**value]**	**Linear range [log**_**2**_**dilution step]**	**Linear range [C**_**q**_**value]**	**Efficiency [%]**
**AZGP1**	40.0	35.6 (34.2 - 36.4)	−13.1 to 0	35.6 to 20.5	81.9 (80.3 - 83.6)
**CALM2**	40.0	35.4 (34.3 - 36.0)	−14.0 to 0	35.4 to 21.6	101.4 (99.8 - 103.2)
**BIRC5**	40.0	36.3 (35.4 - 36.9)	−9.1 to 0	36.3 to 26.7	93.3 (90.5 - 96.2)
**DHCR7**	40.0	36.3 (35.4 - 36.8)	−10.9 to 0	36.3 to 24.5	89.9 (87.6 - 92.3)
**IL6ST**	40.0	36.8 (35.7 - 37.5)	−11.5 to 0	36.8 to 23.3	80.7 (78.8 - 82.7)
**MGP**	40.0	37.2 (35.2 - 38.2)	−13.9 to 0	37.2 to 20.2	76.3 (74.4 - 78.2)
**OAZ1**	40.0	36.6 (35.5 - 37.2)	−12.9 to 0	36.6 to 22.6	89.0 (87.6 - 90.4)
**RBBP8**	40.0	35.6 (34.7 - 36.1)	−9.4 to 0	35.6 to 25.9	96.3 (93.2 - 99.6)
**STC2**	40.0	35.1 (34.2 - 35.7)	−9.9 to 0	35.1 to 24.0	85.2 (82.6 - 87.9)
**UBE2C**	40.0	36.0 (34.9 - 36.7)	−10.1 to 0	36.0 to 24.4	83.3 (81.1 - 85.7)
**RPL37A**	40.0	36.0 (34.5 - 36.7)	−16.4 to 0	36.0 to 19.0	94.5 (92.9 - 96.1)
**HBBV2**	40.0	35.3 (33.6 - 36.2)	−7.6 to 0	35.3 to 25.9	75.4 (70.1 - 81.6)

95% confidence intervals are indicated in brackets. For linear range a maximum deviation from linearity of 1 Ct value was accepted.

All 11 RNA-specific assays were linear up to dilutions between 2^-9^ and 2^-16^ corresponding to C_q_ values between 35.1 and 37.2 (Table 1; supplemental data Figure 2 [see Additional file 1]). Amplification efficiencies ranged from 76% to 101% with a mean efficiency of 88% (Table 1). The DNA-specific *HBB* PCR assay was linear up to a C_q_ value of 35.3 (dilution: 2^-8^) and had an efficiency of 75%.

**Figure 2:**
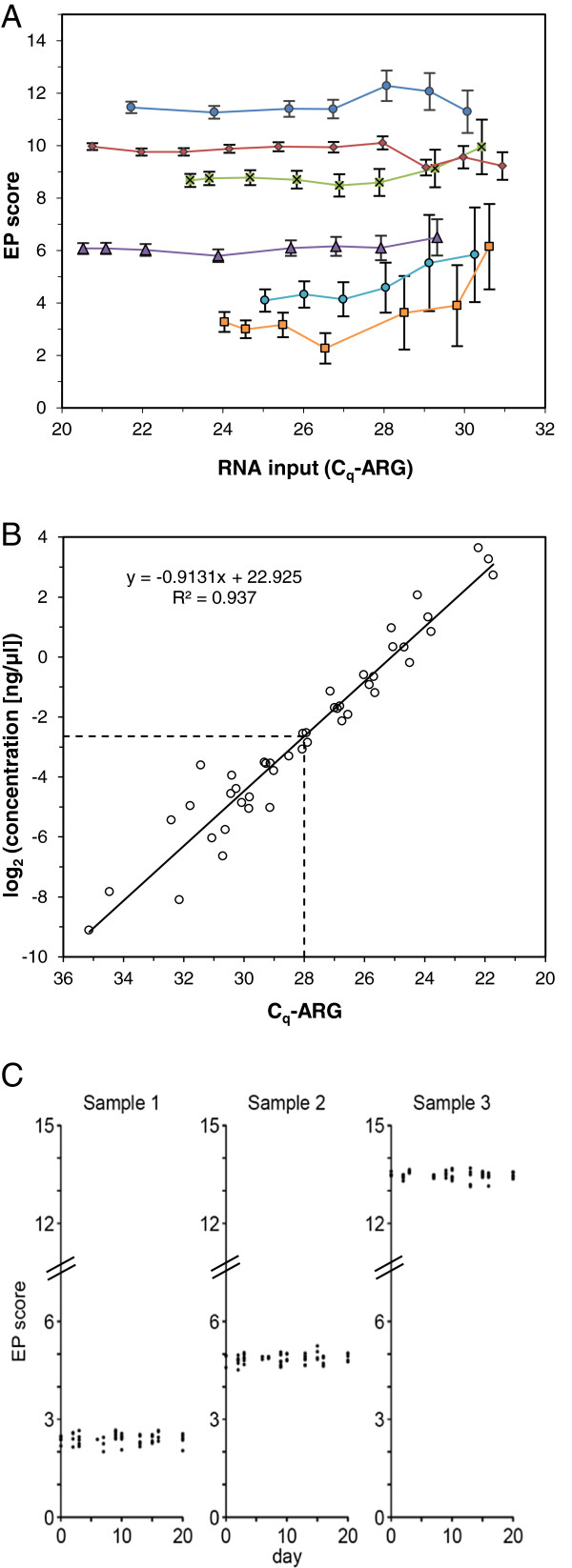
**RNA input range and reproducibility of EndoPredict.** (**A**) EP scores depending on amount of input RNA. RNAs from 6 different FFPE samples were diluted and EP scores were assessed dependent on RNA input (C_q_-ARG as surrogate marker). 95% confidence intervals (CI) of EP scores calculated from the noise model are indicated. (**B**) Correlation between C_q_-ARG and total RNA concentration assessed by RIBOGREEN assay. Lower RNA input limit is indicated by dotted lines. (**C**) Reproducibility of 160 EP scores assessed in three different RNA samples (low risk, close to the decision point, high risk) over time (11 different working days distributed over 21 calendar days). Individual EP measurement results are indicated by dots.

### Input range

For a multigene expression test it is essential to determine the acceptable range of input RNA within which the assay yields accurate results for all variants tested [13]. For that purpose a set of six breast cancer samples with different EP scores ranging from 2.5 to 11.5 were selected. Following RNA isolation different amounts of sample per reaction were assessed by the EndoPredict assay. The average of the C_q_ values of the three reference genes (C_q_-ARG) was used as surrogate for mRNA input. Although an increase of the 95% CI was observed above C_q_-ARGs of 26 the EP score did not significantly change within an RNA input range of C_q_-ARG between 20.5 and 28 (Figure 2A). Analysis of the individual genes showed that *STC2*, *IL6ST*, and *BIRC5* were the first analytes to drop out as the RNA amount was decreased (data not shown). In order to calibrate the C_q_-ARG values to total RNA concentrations the amount of total RNA in a set of 45 samples was assessed (Figure 2B). The range of input RNA concentration without considerable change of the EP score was between 0.16 and 18.5 ng/μl corresponding to a about 100-fold difference.

### Precision

Precision of the multigene expression assay was evaluated under various stipulated operating conditions including day, day time, PCR instrument, position of the sample on the EndoPredict plate, plate lot, reagent lots, and operators and using three different test RNA samples from FFPE breast cancer tissue with a low EP score (2.4), a high EP score (13.5), and an EP score close to the decision point (4.9). In total, 160 EndoPredict tests (Figure 2C) were performed consisting of 5270 measured C_q_ values of the RNA-specific PCR assays and 1280 ΔC_q_ values of the 8 genes-of-interest. The overall variability (standard deviation [SD]) of the EP scores was 0.15 (Table 2), which is 1% of the total EP score range demonstrating robustness and high reproducibility of the test. Interestingly, the major part of the total noise (SD 0.14) was caused by the replicate-to-replicate noise of the PCR assays (repeatability) and was not associated with different operating conditions (reproducibility). The same was true for the variations of C_q_ or ΔC_q_ values which showed overall standard deviations (total noise) of 0.20 and 0.12, respectively (Table 2). Repeatability and reproducibility of the individual gene-specific PCR assays are summarized in supplemental data Tables 1&2 (see Additional file 1).

**Table 2:** **Overall variabilities and variabilities of the EP scores, C**_**q**_**values, and normalized ΔC**_**q**_**values of all genes**

	**Standard deviations**		
	**C**_**q**_	**ΔC**_**q**_	**EP**
**variables**			
day	0.024	0.013	0.006
day time	< 0.001	0.016	<0.001
PCR instrument	0.027	0.009	0.037
sample position	0.028	0.015	<0.001
plate lot	0.014	0.011	<0.001
reagent lot	0.085	0.027	<0.001
operator	0.003	0.014	0.043
**total variable noise**	**0.097**	**0.042**	**0.057**
**replicate noise**	**0.176**	**0.111**	**0.136**
**total noise**	**0.201**	**0.119**	**0.147**

Different operating conditions (variable noise, replicate noise, and total noise) are indicated as standard deviations of the C_q_ values, **Δ**C_q_ values, and the EP scores.

### Verification of performance characteristics in an independent laboratory

The performance characteristics of the EndoPredict assay were verified in a routine laboratory at the Charité in Berlin to confirm that the test performs to specifications also in a routine diagnostic laboratory. The parameters verified were efficiency of the single PCR assays, precision, input range, and analytical accuracy with respect to reference values.

**Figure 3:**
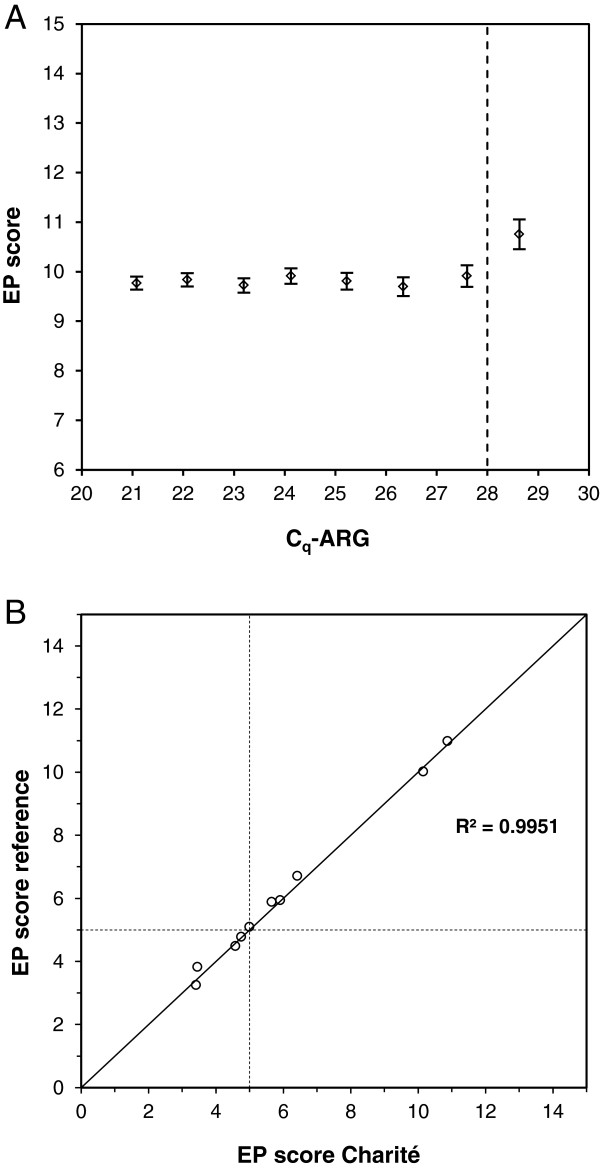
**Verification of performance data at Charité.** (**A**) EP scores depending on amount of input RNA assessed as C_q_-ARG. Pre-specified input limit is indicated by a dotted line. 95% CI are given. (**B**) Correlation of EP scores of 10 different breast cancer FFPE samples assessed at the molecular-pathological laboratory at the Charité compared to reference values assessed at Sividon. The cutoff value between low and high risk for distant metastasis is indicated by dotted lines.

For assessment of linear range and efficiency two independent series of seven 1:2 dilutions of the reference RNA pools and DNA from FFPE tissue were generated. Each nucleic acid concentration was assessed four times. The 11 RNA-specific assays were linear over the whole range of concentrations analyzed (dilutions up to 2^-7^), the *HBB* assay up to dilution step 2^-6^ (Table 3). On average the efficiencies of the RNA assays were 84% and ranged from 78% to 98% which was within the pre-specified reference limits (Table 3). The efficiency of the *HBB* assay was 79%. Assessing the EP scores in each dilution step showed stable values down to an input RNA of C_q_-ARG of 28 verifying the results of the studies at Sividon (Figure 3A).

**Table 3:** Verification of linear dynamic range and PCR efficiency at the molecular-pathological laboratory at the Charité

**Gene**	**Linear range [log**_**2**_**dilution step]**	**Linear range [C**_**q**_**value]**	**Efficiency [%]**
**AZGP1**	−7 to 0	28.5 to 20.5	82.7 (79.1 - 86.7)
**CALM2**	−7 to 0	28.7 to 21.5	97.6 (95.7 - 99.7)
**BIRC5**	−7 to 0	34.2 to 26.6	89.7 (86.4 - 93.2)
**DHCR7**	−7 to 0	32.4 to 24.4	83.2 (79.2 - 87.7)
**IL6ST**	−7 to 0	31.7 to 23.3	78.0 (74.4 - 81.9)
**MGP**	−7 to 0	28.9 to 20.0	72.3 (68.1 - 77.2)
**OAZ1**	−7 to 0	30.4 to 22.6	86.7 (83.6 - 90.1)
**RBBP8**	−7 to 0	33.5 to 25.8	87.6 (84.4 - 91.0)
**STC2**	−7 to 0	32.1 to 23.9	81.2 (77.7 - 85.0)
**UBE2C**	−7 to 0	32.7 to 24.3	78.1 (74.4 - 82.2)
**RPL37A**	−7 to 0	26.6 to 18.7	85.3 (83.1 - 87.5)
**HBBV2**	−6 to 0	32.7 to 25.6	79.0 (74.7 - 83.9)

95% confidence intervals are indicated in brackets. For linear range a maximum deviation from linearity of 1 Ct value was accepted.

Moreover, precision of the EndoPredict test was verified assessing the impact of the day, day time, the position of the sample on the 96-well EndoPredict plate, the reagent lot, and the operator on the reproducibility of the assay. The two RNAs from the tumors with the low and the high EP score were analyzed using the EndoPredict test resulting in 659 C_q_ values, 160 ΔC_q_ values and 20 EP scores. The total variation (standard deviation) of the EP scores was 0.18 (Table 4) and thus almost identical to the variation of the EP scores generated at Sividon (Table 2). In the Charité laboratory variable noise induced by operating conditions had a similar impact on total noise as replicate noise. Standard deviations of C_q_ values and ΔC_q_ values were 0.24 and 0.14 and therefore similar to those at Sividon.

**Table 4:** **Verification of overall variability and variability of the EP scores, C**_**q**_**values, and normalized ΔC**_**q**_**values at the molecular-pathological laboratory at the Charité**

	**Standard deviations**		
	**C**_**q**_	**ΔC**_**q**_	**EP**
**variables**			
day	0.034	0.032	0.093
day time	0.062	0.073	0.074
sample position	<0.001	0.016	<0.001
reagent lot	0.182	0.031	<0.001
operator	0.035	0.073	0.041
**total variable noise**	**0.198**	**0.114**	**0.125**
**replicate noise**	**0.131**	**0.085**	**0.126**
**total noise**	**0.238**	**0.142**	**0.178**

Different operating conditions (variable noise, replicate noise and total noise) are indicated as standard deviations of the C_q_ values, **Δ**C_q_ values, and the EP scores.

Finally, the analytical accuracy of the EndoPredict assay performed in the Charité laboratory was examined. For that purpose, ten breast tumor samples were selected and the EP scores were determined at Sividon. These pre-determined scores ranging from 3.3 to 11.0 were used as reference values. Five of the cases were very close to the predefined cutoff of the EP score. The pre-specified aim for the verification study at Charité was that the difference between the EP score at Charité and the reference EP score was below 1.0 EP units for at least 9 of 10 samples. Charité received a 10 μm tissue section of each of the ten tumors, isolated RNA and performed the EndoPredict test. The aim of this verification study was achieved as the largest deviation from the reference value was 0.36 score units with a mean deviation of 0.15 (Figure 3B). Using the cutoff value of 5 to classify a sample in low or high risk of distant metastasis the concordance of classifications between Charité and Sividon was 90%. The discrepant sample was very close to the cutoff value (EP scores 5.04 vs 4.99). Moreover, an excellent Pearson correlation coefficient of 0.995 (R^2^) was found.

## Discussion

In this study, we showed by means of a defined analytical validation and verification process developed according to the CLSI guidelines that the RT-qPCR-based EndoPredict multianalyte gene expression test is a robust test that can be performed reproducibly and accurately. The resulting performance characteristics therefore meet the requirements needed for a diagnostic test. Moreover, we verified that a comparable performance with respect to assay efficiency, precision, and accuracy can also be achieved in a routine molecular diagnostic laboratory. In addition, this study provides the specifications for analytical verification of EndoPredict in molecular pathological laboratories.

Successful clinical validation of the EndoPredict score in two large clinical trials was published previously [1] resulting in a level of evidence of 1B according to the classification for prognostic biomarkers that has been proposed by Simon et al. [22]. The clinical validation studies which were performed within a prospective-retrospective design showed that the test predicted distant metastasis in patients with primary ER-positive, HER2-negative breast cancer and provided significant prognostic information beyond standard parameters to assess the risk of metastasis. Both, clinical and analytical validations of the EndoPredict multigene assay now fulfill the recently published recommendations for translating omics-based tests from research laboratory to clinical practice [23].

The difficulty of a comprehensive analytical validation of a highly complex genomic multianalyte assay such as EndoPredict is that there are no evaluation guidelines to cover all relevant aspects. Therefore, we specified a validation program for both, the 12 single analyte assays and the combined multigene assay. Where applicable, established guidelines of the CLSI as well as the MIQE (Minimum Information for Publication of Quantitative Real-Time PCR Experiments) guidelines were followed [13,18]. Therefore, the analytical validation process conformed to the standard assay validation formats as close as reasonably possible.

Besides EndoPredict, other prognostic multigene expression tests for patients with breast or other cancers like MammaPrint [8], Oncotype DX Breast Cancer or Colon Cancer Assays [9,10], or PAM50 [11,12] are commercially available. Analytical performance characteristics of these tests, which must be performed in reference laboratories, were published only for some tests such as MammaPrint [24] or the two Oncotype DX assays [25,26]. Here, we assessed the analytical performance data of EndoPredict and provide further evidence that EndoPredict is suitable for decentralized testing in specialized molecular pathological laboratories which is a unique feature not shown for the other RNA-based multigene expression tests. On one hand this conclusion is supported by the verification of the performance characteristics and the accuracy of the measurements in a routine laboratory. On the other hand, the data of the precision study in two different laboratories showed a total variation (standard deviation) of the EP score of 0.15 and 0.18, respectively, which is about 1% of the total EP score range from 0 to 15. This is similar to the variation of the Oncotype DX Breast Cancer Assay performed in one centralized company laboratory [25]. Interestingly, the main factor influencing total noise was not one of the individual variables tested, such as operator, PCR machine, day, day-time, or reagent lots but rather replicate-to-replicate noise which is assumed to be caused by PCR technique-inherent noise. In order to account for PCR-inherent noise the EndoPredict algorithm includes a “noise model” estimating the acceptable variance of replicate noise with respect to the C_q_ value. On this basis outlier elimination is accomplished [1]; one of its principal requirements is triplicate measurements.

Robust performance of the EndoPredict test in a decentralized setting was also shown in a successful prospective proficiency testing study including seven different molecular pathology laboratories [2]. In this study, 69 out of 70 EndoPredict measurements were within the pre-specified range, 100% of the samples were correctly classified as low or high risk of metastasis, and the total variation (standard deviation) of all measurements was 0.25 units, corresponding to 1.7% of the whole range of the EP score. The slightly larger total variation in comparison to the results from the analytical precision study where large RNA pools were used might be due to the fact that in the proficiency testing the participants received tissue sections and not RNA for testing, thus including variation induced by tumor heterogeneity.

A recent study assessed the variability of Ki-67 immunohistochemistry, which is a standard antibody-based diagnostic test in pathology used for treatment decision making in luminal breast cancer [27]. The authors found standard deviations of Ki-67 results obtained by 15 pathologists on centrally stained slides of three breast carcinomas ranging from 21.7% to 24.1%. Interestingly, even clear guidelines how to assess Ki-67 could not improve variability.

Although our results might suggest a higher reproducibility of the PCR-based test using standardized instruments and reagents, it is important to know that the pathological laboratories involved in this technical verification study as well in the proficiency testing of EndoPredict were highly experienced in molecular work. Therefore, the results might be different in laboratories with less molecular diagnostic experience and ongoing quality control by periodical round robin tests might be reasonable.

A critical issue for accurate results from any diagnostic assay is the use of optimal sample material. For this validation study optimal FFPE tumor material was selected by experienced pathologists on the basis of adjacent HE-stained tissue slides. Hence, in order to obtain high-quality EndoPredict results an expert pathological evaluation of the tissue specimens is mandatory. Moreover, a highly standardized method for RNA isolation as used in this validation study and in the EndoPredict proficiency testing is necessary. In this study, the validation of the RNA extraction method was not a primary aim. The silica-coated magnetic beads-based method used and recommended for EndoPredict was thoroughly validated in previous studies showing a reliable analysis of RNA transcript levels by RT-qPCR in FFPE tissue [4-6]. Finally, the PCR platform used in this validation study was validated and CE-marked for diagnostic purposes by the manufacturer. It also supports robust performance of the EndoPredict assay. Therefore, change of isolation method, enzymes, and PCR platform might alter performance characteristics of this multianalyte assay.

## Conclusions

In conclusion, this study provides the analytical performance characteristics of the EndoPredict breast cancer prognosis multigene expression assay. They can be used as a reference for analytical verification of the test in molecular pathological laboratories. Moreover, the study shows a robust and reliable performance of the test and provides conclusive evidence that RT-qPCR-based quantitative multigene expression analysis of FFPE tissue samples is feasible in a decentral setting in molecular pathological laboratories. This is a major technical advance in comparison with existing prognostic multigene expression tests which are performed in central reference laboratories. Together with the clinical validation studies and the prospective analytical proficiency testing program with seven different molecular pathological laboratories these results provide the basis for the application of EndoPredict as a test to assess prognosis under endocrine therapy in clinical decision making in a decentralized environment.

## Abbreviations

EP: Endopredict; ER: Estrogen receptor; FFPE: Formalin-fixed, paraffin-embedded; RT-qPCR: Reverse transcription quantitative real-time PCR; LoD: Limit of detection; C_q_: Quantification cycle; GOI: Gene-of-interest; LoB: Limit of blank; C_q_-ARG: Average C_q_ value of reference genes; CI: Confidence interval.

## Competing interests

RK, KB, FH, CR, MA, TR, CW, JCB, KEW, KF, CP are employees of Sividon Diagnostics GmbH, RK, KB, CR, MA, TR, CW, KEW, CP, MD, CD hold shares of Sividon Diagnostics GmbH. MS, MF, PD received speakers’ honoraria from Sividon Diagnostics. Sividon Diagnostics has applied for a patent relating to the content of the manuscript.

## Author’s contributions

RK, KB, JCB, KEW, MS, MF, PD, CP, MD, CD were involved in conception and design of the study as well as in analysis or interpretation of data. JP, BVS, FH, CR, MA, TR, CW, KF, BMM acquired, analyzed and interpreted data. All authors have been involved in drafting or critically revising the manuscript and approved the manuscript.

## Pre-publication history

The pre-publication history for this paper can be accessed here:

http://www.biomedcentral.com/1471-2407/12/456/prepub

## Supplementary Material

Additional file 1 **Supplemental Data:** File contains detailed descriptions of methods as well as supplemental tables and figures which could not be included in the main manuscript. Click here for file
