# Supplementary material for: Decentral gene expression analysis: analytical validation of the Endopredict genomic multianalyte breast cancer prognosis test
Source: BMC Cancer. 2012 Oct 5;12:456. doi: 10.1186/1471-2407-12-456 (PMC3534340; doi:10.1186/1471-2407-12-456)
Supplement: Additional file 1 — Supplemental Data: File contains detailed descriptions of methods as well as supplemental tables and figures which could not be included in the main manuscript. [file 1471-2407-12-456-S1.pdf]

Supplemental Data

Reference and testing material

For assessment of the limit of detection (LoD), linear range, and efficiency of the single PCR assays sample pools with high concentrations of the RNA species assayed in the EndoPredict were required. Since the genes are differentially expressed and their expression strength is associated with either good or bad prognosis no single tumor sample could be identified with a high-enough gene expression of all genes to be tested. Therefore, as recommended by CLSI guideline MM16-A a large pool of control RNA was generated to be repeatedly analyzed with consistent results [1]. For that purpose, anonymized residual FFPE tumor specimens from 20 breast cancer tumors were screened by the EndoPredict test for gene expression levels of the respective genes. As a result, a pooled reference RNA preparation (1:1:1 mixture) was generated from total RNA isolated from one EP low risk and two EP high risk tumors. Before pooling, RNA was isolated as described in material and methods of the main paper. For the precision studies, three tumor specimens were selected: one tumor classified by the EP as low risk, one tumor classified as high risk and one tumor close to the decision point of EP between low and high risk. For the correlation study between the two laboratories, ten tumor samples were chosen with EP scores spanning almost the whole score range.

For analytical validation of the *HBB*-DNA-specific PCR assay, total DNA was isolated from one FFPE breast cancer tumor specimen as described in the material and methods section of the main paper.

Breast tumors used as reference samples were fixed with neutral, phosphate-buffered 10% formalin and paraffin embedded using standard protocols. FFPE blocks were stored at room temperature. An invasive tumor content of at least 30% was confirmed by hematoxilin / eosin-stained tissue sections adjacent to those sections used in this study.

A standardized reference RNA (Stratagene qPCR Human Reference Total RNA, Agilent Technologies) was tested for each gene on each plate to serve as a positive control of the

RT-qPCR assays. Human Genomic DNA (Roche Applied Bioscience) was used as a positive control for the *HBB*-DNA PCR assay.

Assessment of limit of blank (LoB), limit of detection (LoD), and linear dynamic range. LoB was defined as the 5%-percentile of the distribution of C_q-values measured in a blank sample without analyte [2]. 187 blank measurements were performed for each of the 11 RNA-PCR assays and 104 of the *HBB* assay (60 measurements are recommended as a minimum). LoB was calculated for an α=5% as described in NCCLS EP17-A [3]. Negative PCR results were set to C_q value 40.

For assessment of LoD, linear dynamic range, and PCR efficiency four independent series of 20 serial gravimetrically controlled 1:2 dilutions ($\log_2$) were generated from a pooled RNA sample (DNA sample for *HBB* PCR) from FFPE tissue resulting in 21 different concentrations (3-5). Dilutions were performed by different operators at different days to reflect an analytical variation. Each dilution step was assessed between 16 and 40 times (dependent on dilution step and gene) for the 12 different analytes resulting in at least 60 replicates of low concentration samples as recommended for assessment of LoD [3].

LoD was defined as the smallest amount of the reference RNA at which the C_q value is below the LoB with a probability of 95%. An absolute quantification of the 11 different target sequences in total RNA from FFPE tumors was not possible. Accordingly, LoD was referred to the fold-dilution of the reference RNA and to the respective C_q value as a surrogate for the amount of the individual analytes. For assessment of the LoD, relative frequencies of C_q values $f_r = N_{Cq<LoB} \,/\, N$ ($qN_{Cq<LoB}$: number of C_q values below LoB, N: total number of C_q values) were determined for each $\log_2$ dilution step d (see supplemental data figure 1, blue) and a binomial regression model was calculated using the logistic function $\mathrm{f}(d) = 1/(1 + \exp(-(c_1 + c_2\, d)))$ (see supplemental data figure 1, green; c_1, c_2: regression coefficients). The LoD was then calculated from this model (see supplemental data figure 1, red). The respective C_q value, $C_q(LoD)$, was estimated from the linear regression model derived during the linearity analysis (see below).

The linearity of the quantitative assays was evaluated as described in NCCLS EP6-A [4,5]. Since concentrations of the individual mRNA specimen in the total RNA preparation were not known, the linearity of the C_q values was assessed for each corresponding dilution step up to LoD (see supplemental data figure 2). Within this dilution step range a C_q value was considered as a statistical outlier and excluded from analysis if the value of the Studentized residual was outside the 99.5% central interval of the Student's t-distribution of the corresponding dilution step. Next, regression models with 1st (linear), 2nd (quadratic), and 3rd order (cubic) polynomial functions were fitted to the C_q value measurements using log_2 dilution values. It was tested if the non-linear polynomial coefficients were significantly different from zero (p=0.05). If none of the non-linear coefficients were significant, the gene was considered linear. Otherwise, the model fitting the data best (smallest mean squared error) was compared to the linear model by calculating the absolute difference between these models at the examined log_2 dilutions. If this deviation from linearity did not exceed 1 C_q value, the gene was considered linear. If not, the dilution step range was reduced and the regression analysis was repeated.

Assessment of precision by ANOVA.

Before analysis of variability statistical outliers were identified and removed within C_q value triplicates (those sets of replicates being combined into one EP score value: on the same plate, same position, …) based on a pre-defined independent "noise model" that estimates the variance of replicate-to-replicate as described in Filipits et al. [6]. Univariate N-way analysis of variance (ANOVA) was used to divide the total noise (reproducibility standard deviation) into noise of variables (standard deviations between variable values) and the residual noise (repeatability standard deviation), which is assumed to reflect the replicate-to-replicate variation. The standard deviations were estimated using the variance component estimates of an ANOVA model with random effects as described in ISO 5725-2 [7]. The variable noise (variance between the groups) summarizes the variance components of all variables, and the total noise includes both the variable and replicate noise.

References.

1. CLSI. **Use of external RNA controls in gene expression assays; approved guideline. CLSI document MM16-A.** Wayne, PA: Clinical and Laboratory Standards Institute; 2006.

2. CLSI. **Verification and validation of multiplex nucleic acid assays; approved guideline. CLSI document MM17-A.** Wayne, PA: Clinical and Laboratory Standards Institute; 2008.

3. NCCLS. **Protocols for determination of limits of detection and limits of quantitation; approved guideline.** NCCLS document EP17-A. Wayne, PA: NCCLS; 2004.

4. NCCLS. **Evaluation of the Linearity of Quantitative Measurement Procedures: A Statistical Approach; Approved Guideline.** NCCLS document EP6-A. Wayne, PA: NCCLS; 2003.

5. Bustin SA, Benes V, Garson JA, Hellemans J, Huggett J, Kubista M, Mueller R, Nolan T, Pfaffl MW, Shipley GL *et al*: **The MIQE guidelines: minimum information for publication of quantitative real-time PCR experiments**. *Clin Chem* 2009, **55**(4):611-622.

6. Filipits M, Rudas M, Jakesz R, Dubsky P, Fitzal F, Singer CF, Dietze O, Greil R, Jelen A, Sevelda P *et al*: **A new molecular predictor of distant recurrence in ER-positive, HER2-negative breast cancer adds independent information to conventional clinical risk factors**. *Clin Cancer Res* 2011, **17**(18):6012-6020.

7. ISO 5725-2. **Accuracy (trueness and precision) of measurement methods and results – Part 2: basic method for the determination of repeatability and reproducibility of a standard measurement method. British Standards Institution BS ISO 5725-2:1994.**

Supplemental Table 1. Overall variability and variability of the C_q values for the individual EndoPredict PCR assays assessing RNA levels induced by the different operating conditions (variables). Variable noise, replicate noise, and total noise are indicated as standard deviations of the C_q values.

	standard deviations										
	**AZGP1**	**CALM2**	**BIRC5**	**DHCR7**	**IL6ST**	**MGP**	**OAZ1**	**RBBP8**	**STC2**	**UBE2C**	**RPL37A**
**variables**											
day	0.022	0.028	0.036	0.025	0.021	<0.001	0.018	0.010	0.011	0.032	0.023
day time	<0.001	<0.001	0.020	<0.001	<0.001	<0.001	0.023	<0.001	<0.001	<0.001	0.011
PCR instrument	0.026	0.017	0.052	0.017	0.031	0.026	0.022	0.059	0.050	0.062	0.026
sample position	0.053	0.026	0.052	0.013	0.031	0.022	<0.001	0.010	0.039	0.014	0.021
plate lot	0.023	0.014	0.043	0.005	0.015	<0.001	0.007	0.015	0.030	<0.001	0.009
reagent lot	0.111	0.068	0.160	0.055	0.087	0.098	0.078	0.085	0.065	0.068	0.047
operator	0.007	<0.001	0.088	<0.001	<0.001	<0.001	0.014	<0.001	<0.001	<0.001	<0.001
**total variable noise**	**0.129**	**0.081**	**0.205**	**0.064**	**0.101**	**0.103**	**0.087**	**0.106**	**0.097**	**0.098**	**0.064**
**replicate noise**	**0.120**	**0.117**	**0.247**	**0.138**	**0.163**	**0.184**	**0.167**	**0.199**	**0.187**	**0.208**	**0.095**
**total noise**	**0.176**	**0.143**	**0.321**	**0.153**	**0.191**	**0.211**	**0.189**	**0.226**	**0.211**	**0.230**	**0.115**

Supplemental Table 2. Overall variability and variability of the ΔC_q values normalized to *CALM2*, *OAZ1* and *RPL37A* for the PCR assays assessing EndoPredict RNA levels induced by the different operating conditions (variables). Variable noise, replicate noise, and total noise are indicated as standard deviations of the ΔC_q values.

	**standard deviations**							
	**AZGP1**	**BIRC5**	**DHCR7**	**IL6ST**	**MGP**	**RBBP8**	**STC2**	**UBE2C**
**variables**								
day	0.016	0.010	<0.001	0.011	0.009	0.012	<0.001	0.018
day time	0.018	0.036	0.017	0.009	<0.001	0.021	<0.001	<0.001
PCR instrument	0.008	0.051	0.015	0.009	0.011	0.043	0.034	0.055
sample position	0.036	0.035	<0.001	0.013	<0.001	<0.001	0.023	<0.001
plate lot	0.013	0.032	<0.001	<0.001	0.014	0.010	0.037	<0.001
reagent lot	0.045	0.095	0.001	0.020	0.032	0.017	<0.001	<0.001
operator	0.017	0.093	<0.001	<0.001	<0.001	<0.001	<0.001	<0.001
**total variable noise**	**0.067**	**0.155**	**0.022**	**0.030**	**0.038**	**0.053**	**0.055**	**0.058**
**replicate noise**	**0.062**	**0.156**	**0.076**	**0.092**	**0.100**	**0.099**	**0.080**	**0.124**
**total noise**	**0.091**	**0.220**	**0.079**	**0.096**	**0.107**	**0.113**	**0.097**	**0.137**

Supplemental figures

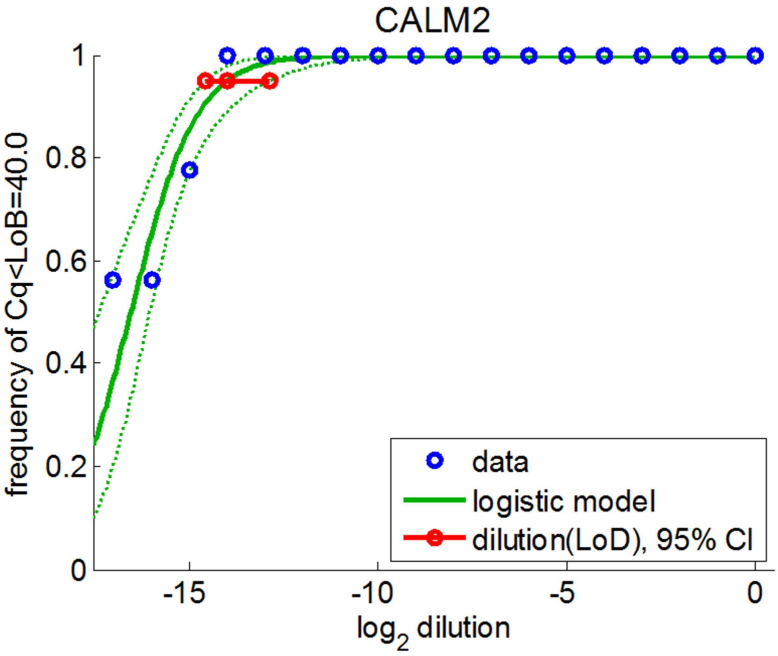

Supplemental figure 1. Estimation of the limit of detection (LoD): The LoD was defined as the concentration of reference RNA ($\log_2$ dilution) at which the C_q value is below the limit of blank (LoB) with a probability of 95%. For detailed description of calculations as well as of the color coding see paragraph “Assessment of limit of blank (LoB), limit of detection (LoD), and linear dynamic range” above. Analysis for a representative PCR assay (*CALM2*) is shown.

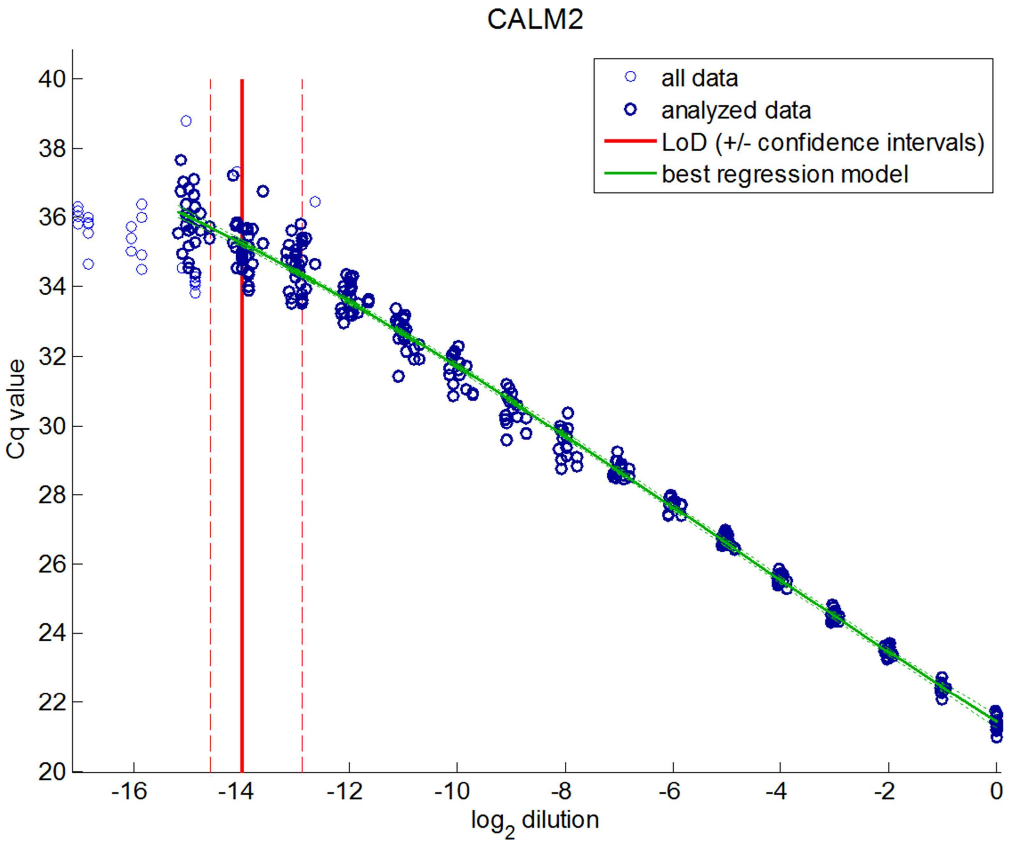

Supplemental figure 2. Estimation of the linear dynamic range: The linearity of the C_q values was assessed down to the limit of detection (LoD). The gene was considered linear if the deviation from linearity, i.e. the difference between the best and the linear regression model, did not exceed 1 C_q value. Details see paragraph “Assessment of limit of blank (LoB), limit of detection (LoD), and linear dynamic range” above. Analysis for a representative PCR assay (*CALM2*) is shown.
